# Transgenic rice expressing *Allium sativum *leaf agglutinin (ASAL) exhibits high-level resistance against major sap-sucking pests

**DOI:** 10.1186/1471-2229-8-102

**Published:** 2008-10-14

**Authors:** Bharathi Yarasi, Vijayakumar Sadumpati, China Pasalu Immanni, Dasavantha Reddy Vudem, Venkateswara Rao Khareedu

**Affiliations:** 1 Centre for Plant Molecular Biology, Osmania University, Hyderabad, 500 007, India; 2 Directorate of Rice Research, Rajendranagar, Hyderabad, 500030, India

## Abstract

**Background:**

Rice (*Oryza sativa*) productivity is adversely impacted by numerous biotic and abiotic factors. An approximate 52% of the global production of rice is lost annually owing to the damage caused by biotic factors, of which ~21% is attributed to the attack of insect pests. In this paper we report the isolation, cloning and characterization of *Allium sativum *leaf agglutinin (*asal*) gene, and its expression in elite indica rice cultivars using *Agrobacterium*-mediated genetic transformation method. The stable transgenic lines, expressing ASAL, showed explicit resistance against major sap-sucking pests.

**Results:**

*Allium sativum *leaf lectin gene (*asal*), coding for mannose binding homodimeric protein (ASAL) from garlic plants, has been isolated and introduced into elite indica rice cultivars susceptible to sap-sucking insects, viz., brown planthopper (BPH), green leafhopper (GLH) and whitebacked planthopper (WBPH). Embryogenic calli of rice were co-cultivated with *Agrobacterium *harbouring pSB111 super-binary vector comprising garlic lectin gene *asal *along with the herbicide resistance gene *bar*, both under the control of CaMV35S promoter. PCR and Southern blot analyses confirmed stable integration of transgenes into the genomes of rice plants. Northern and western blot analyses revealed expression of ASAL in different transgenic rice lines. In primary transformants, the level of ASAL protein, as estimated by enzyme-linked immunosorbent assay, varied between 0.74% and 1.45% of the total soluble proteins. *In planta *insect bioassays on transgenic rice lines revealed potent entomotoxic effects of ASAL on BPH, GLH and WBPH insects, as evidenced by significant decreases in the survival, development and fecundity of the insects.

**Conclusion:**

*In planta *insect bioassays were carried out on *asal *transgenic rice lines employing standard screening techniques followed in conventional breeding for selection of insect resistant plants. The ASAL expressing rice plants, bestowed with high entomotoxic effects, imparted appreciable resistance against three major sap-sucking insects. Our results amply demonstrate that transgenic indica rice harbouring *asal *exhibit surpassing resistance against BPH, GLH and WBPH insects. The prototypic *asal *transgenic rice lines appear promising for direct commercial cultivation besides serving as a potential genetic resource in recombination breeding.

## Background

Globally, more than 3 billion people from Asia and other countries depend on rice (*Oryza sativa*, L.) as their staple food, and by 2025 about 60% more rice must be produced to meet the needs of the growing population [1]. Productivity losses resulting from herbivorous insects have been estimated between 10–20% for major crops grown worldwide [2]. Rice productivity is adversely impacted by numerous biotic and abiotic factors. An approximate 52% of the global production of rice is lost annually owing to the damage caused by biotic factors, of which ~21% is attributed to the attack of insect pests [3]. Furthermore, insects are known to show widespread occurrence with wide variation in their intensity and feeding habits. Insects belonging to Delphacidae and Cicadellidae contain a large group of sap-sucking planthoppers and leafhoppers, respectively. As such, they are difficult to control and manage, resulting in huge yield losses occurring in most of the rice growing areas. Insects not only cause direct losses to the agricultural produce but also act as vectors for various plant pathogens [4,5]. Three major sap-sucking pests of rice, viz., brown planthopper (*Nilaparvata lugens*, BPH), green leafhopper (*Nephotettix virescens*, GLH) and whitebacked planthopper (*Sogatella furcifera*, WBPH) are known to cause severe damage to the rice plant besides acting as vectors for major viral diseases.

Although chemical control of insect pests is an effective option, most often it is expensive and depends mainly on the weather conditions. Extensive application of chemical pesticides not only builds up resistance in insect pests but also proves deleterious to the beneficial organisms such as pollinators, nutrient cyclers and natural pest-controlling agents owing to their non-selective properties. Moreover, indiscriminate usage of pesticides exerts harmful effects on the environment and human health through food chain. As such, adoption of insect resistant cultivars has been considered as the most economic and eco-friendly strategy for pest management. Genetic enhancement of rice through conventional methods is often constrained by narrow gene pools besides strong barriers to crossability. In this context, transgenic technology can be adopted as an alternative approach for evolvement of insect resistant varieties by introducing exotic resistance genes into leading rice cultivars.

In recent times, successful attempts have been made to prospect for novel candidate genes that convey tangible resistance against major insect pests from various sources such as microbes, plants and animals. Different versions of *Bacillus thuringiensis *endotoxin encoding genes (*cry*) have been introduced into diverse crop plants to protect against the damages caused by lepidopteran and coleopteran insects which feed by chewing [6-12]. Furthermore, plants are known to serve as sources of non-*Bt *insecticidal proteins such as lectins, protease inhibitors as well as ribosome-inactivating proteins [13]. However, protease inhibitors showed little success in controlling major sap-sucking pests [14]. Recent reports provide detailed accounts on the economic, environmental and health benefits of various insect-resistant transgenic crops [15,16].

Lectins are carbohydrate-binding proteins that specifically recognize diverse sugar structures and thus mediate various biological processes, viz., cell-cell and host-pathogen interactions, and serum glycoprotein turnover besides innate immune responses [17]. Lectins are known to occur in most of the organisms ranging from viruses and bacteria to plants and animals [18]. They represent a heterogenous group of oligomeric proteins that vary widely in size, structure and molecular organization besides constitution of combining sites with respective receptors on gut epithelial cells of insects. The possible mechanism of lectin toxicity in insects seems to involve the binding of lectin to the gut surface, leading to local lesions in the gut [19]. The insecticidal activity of carbohydrate-binding plant lectins against insects belonging to coleoptera, diptera, lepidoptera and homoptera have been amply investigated [20-25].

A wide range of lectins exhibiting either mannose or mannose/glucose sugar binding affinity, including *Galanthus nivalis *agglutinin (GNA), Concanavalin A (Con A) and *Pisum sativum *agglutinin (PSA), revealed palpable antimetabolic effects towards members of the homopteran insects both under *in vitro *[26-28] as well as *in planta *conditions [27,29,30]. Among the mannose-binding lectins, *G. nivalis *agglutinin (GNA) has been widely studied and introduced into different plants, viz., rice, wheat and tuber crops [20,31,32,23,24]. Transgenic plants expressing GNA showed significant entomotoxic effects as evidenced by insect bioassays under controlled conditions [33,31,30-32,5,35,23,24]. Similarly, bioassays based on artificial-diet-feeding system, using mannose-specific lectin from *Allium sativum *agglutinin (ASA), showed antimetabolic effects towards BPH and GLH insects [29,25]. Expression of garlic lectins, ASAL and ASA-II in tobacco conferred resistance against tobacco aphid and cotton leaf worm, respectively [36,37]. Saha et al. [38] reported that transgenic rice lines expressing garlic lectin gene (*asal*) exhibit increased resistance against GLH and BPH pests. In three subspecies of rice, significant advances made in the regeneration protocols and gene delivery methods have facilitated introduction of beneficial genes for various agronomic traits. In the recent past, it has been established that *Agrobacterium*-mediated transformation is an efficacious method for transferring novel candidate genes into elite indica rice varieties [39-41,23,12].

The present investigation deals with the isolation, cloning and characterization of lectin (*asal*) gene from *A. sativum*, and its expression in elite indica rice cultivars using *Agrobacterium*-mediated genetic transformation method. Molecular evidences suggest stable integration of *asal *and *bar *genes into the genomes of rice plants, and their variable expression at RNA and protein levels. The stable transgenic lines, expressing ASAL, showed explicit resistance against major sap-sucking insects, viz., BPH, GLH and WBPH.

## Results

The observations described herein pertain to the isolation of *asal *gene from garlic plants and its overexpression in two elite indica rice cultivars. The presence and expression of transgenes (*asal *and *bar*) in rice plants has been demonstrated through Southern, northern and western analyses. The various ASAL-expressing rice lines furnished marked resistance against three major sap-sucking pests, viz., BPH, GLH and WBPH.

### Isolation of A. sativum agglutinin gene (asal) and construction of pSB111-bar-asal plant expression vector

**Figure 1:**
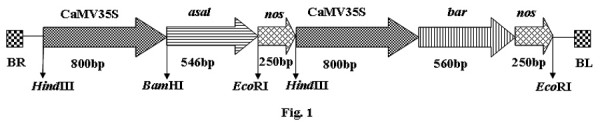
Restriction map of T-DNA region of pSB111 containing *bar *and *asal *expression units.

Coding sequence of the *asal *was isolated from garlic plants through the synthesis of cDNA followed by PCR using gene specific primers. The PCR product contained 546 bp coding sequence (Genbank accession no: DQ525625) that codes for a polypeptide of 181 amino acids (ABF70332), containing a signal peptide of 30 a.a. The ASAL protein showed maximum identity of 98% with the mannose specific lectins isolated from the leaf (AAW48531) and bulbs (AAB64238) of garlic plants. The plant expression cassette, comprising CaMV35S promoter, *asal *and *nos *terminator, was cloned at *Hind*III site of pSB11 *bar *intermediate vector of the *Agrobacterium *containing *bar *gene expression cassette (Fig. 1). The recombinant clone was then introduced into *Agrobacterium *strain LBA4404 by triparental mating and the resultant super-binary vector was designated as pSB111-*bar*-*asal *(Fig. 1).

### Genetic transformation and production of transgenic rice plants

**Figure 2:**
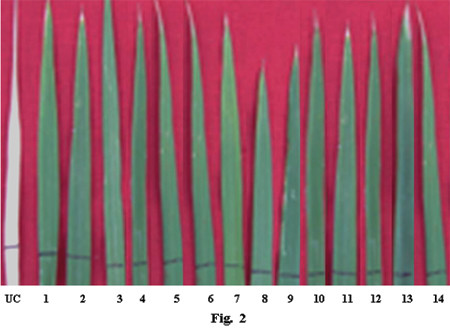
**Basta treated leaves of putative transformants showing complete tolerance to the herbicide**. Lane. UC: Untransformed control plant showing complete damage to the herbicide Basta,. Lanes 1–11: Different transformants of Chaitanya showing herbicide tolerance,. Lanes 12–14: Transformants of BPT5204 showing herbicide tolerance.

To insert *asal *gene into rice plants, embryogenic calli of rice (cvs. Chaitanya and BPT5204) was co-cultivated with the *Agrobacterium *strain LBA4404 harbouring Ti-plasmid pSB111-*bar*-*asal*. A total number of 47 and 29 putative transformants were obtained from 2116 calli of Chaitanya and 4381 calli of BPT5204, respectively. From these, 14 transformants of Chaitanya and 3 of BPT5204 were selected for further analyses based on their high tolerance to herbicide (0.25%) BASTA (Fig. 2).

### Molecular analysis of primary (T_0_) transgenic plants

**Figure 3:**
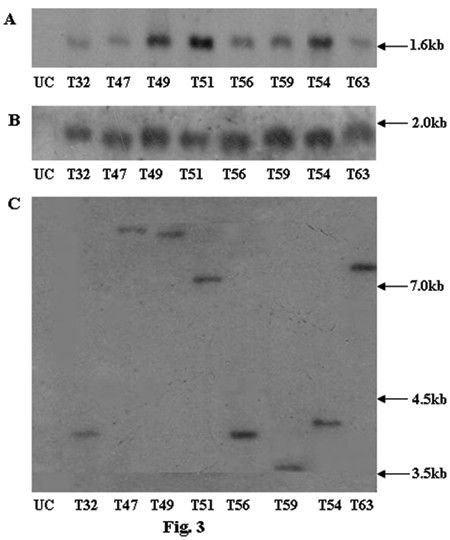
**Southern blot analysis of genomic DNA from the leaves of transgenic rice lines and control plants**. (A) Genomic DNA digested with *Hind*III and probed with *asal *coding sequence; (B) Genomic DNA digested with *Eco*RI and probed with *bar *coding sequence; (C) Genomic DNA digested with *Eco*RI and probed with *asal *coding sequence; Lane UC: DNA from untransformed control plant, Lanes 1–6: DNA from T_32_, T_47_, T_49_, T_51_, T_56_and T_59 _transgenic lines of Chaitanya, Lanes 7–8: DNA from T_54_and T_63 _transgenic lines of BPT5204.

**Figure 4:**
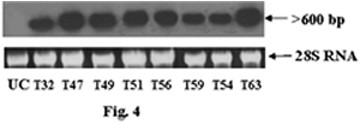
**Northern blot analysis for the expression pattern of *asal *in different transgenic rice lines**. Lane UC: RNA from untransformed control plant, Lanes 1–6: RNA from T_32_, T_47_, T_49_, T_51_, T_56 _and T_59 _transgenic lines of Chaitanya, Lanes 7–8: RNA from T_63 _and T_54 _transgenic lines of BPT5204. Ethidium bromide stained 28S rRNA band is shown under northern blot for amount of RNA loading.

**Figure 5:**
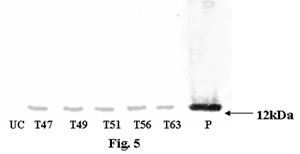
**Western blot analysis of leaf extracts of transgenic lines along with controls**. Lane UC: Protein extract (5 μg) from untransformed control plant, Lanes 1–4: Protein extract (5 μg) from T_47_, T_49_, T_51 _and T_56 _transgenic lines of Chaitanya, Lane 5: Protein extract (5 μg) from T_63 _transgenic line of BPT5204, Lane 6: Purified ASAL protein (50 ng).

Genomic DNA was isolated from the BASTA tolerant transgenic rice plants as well as from the untransformed control plants. PCR analysis of transgenic rice plants showed amplification of 560 bp and 546 bp products, representing *bar *and *asal *coding sequences, while control plants failed to show such amplification (data not shown). Southern blot analysis was carried out using BASTA and PCR positive plants. When genomic DNA of transgenic plants was digested with *Hind*III and probed with *asal *coding sequence, it showed hybridizable band of ~1.6 kb (Fig. 3A). Similarly, *Eco*RI digested DNA of transgenics probed with *bar *sequence showed ~1.9 kb band (Fig. 3B). Genomic DNA of different transgenic plants, digested with *Eco*RI and probed with the *asal*, showed a distinct hybridizable band of >3 kb (Fig. 3C). These bands correspond to the expression units of *bar *and *asal *transgenes introduced into the transgenic rice plants. Conversely, the untransformed control plants failed to show any hybridizable band with both the probes. Northern blot analysis was performed using the RNA from Southern positive plants to assess the expression of *asal *gene in different transgenic rice lines; presence of a >600 bp hybridizable band of varied intensity was visualized in diverse transgenic lines (Fig. 4). Western blot analysis of leaf extracts from transgenic plants showed the presence of a polypeptide of >12 kDa corresponding to the purified *asal *protein when treated with anti-*asal *antibodies (Fig. 5). Whereas, no such protein was observed in the untransformed control plants. The level of ASAL expression in transgenic plants was determined by the enzyme-linked immunosorbent assay (ELISA), and the amount of ASAL among transformants ranged between 0.74% and 1.45% of the total soluble proteins.

### Inheritance pattern of asal and bar genes in T_1 _generation

To investigate the inheritance pattern of the transgenes, selfed seed collected from the primary (T_0_) transformants were germinated and T_1 _progenies were grown to maturity in the glass house. Eight T_1_lines of Chaitanya, viz., T_32_, T_47_, T_49_, T_51_, T_56_, T_59_, T_63 _and T_68_, and three T_1 _lines of BPT5204, viz., T_43_, T_54 _and T_63_, were tested with the herbicide BASTA and were also subjected to insect bioassays. In T_1 _progenies, both the transgenes *bar *and *asal *showed a monogenic segregation of 3 resistant: 1 susceptible plant(s) besides co-segregation in a normal Mendelian fashion for BASTA tolerance as well as for insect resistance (Table. 1). These transgenic lines were healthy and were found similar to that of untransformed control plants for various morphological characters with normal seed fertility.

**Table 1:** Inheritance pattern of transgenes, and insect bioassays for brown planthopper (BPH), green leafhopper (GLH) and whitebacked planthopper (WBPH) in T_1 _generation

T_1 _progenies	Test/Bioassay	No of plants tested	No. of plants resistant	No. of plants susceptible	Segregation ratio	χ 2 value	p-value
T32 Chaitanya	Basta	41	29	12	3:1	0.380	0.537
T47 Chaitanya	Basta	35	19	6	3:1	0.013	0.909
T49 Chaitanya	Basta	29	21	8	3:1	0.096	0.756
T51 Chaitanya	Basta	50	37	13	3:1	0.026	0.871
T56 Chaitanya	Basta	41	29	12	3:1	0.380	0.537
T59 Chaitanya	Basta	40	29	11	3:1	0.130	0.718
T63 Chaitanya	Basta	36	29	7	3:1	0.273	0.601
T68 Chaitanya	Basta	27	20	7	3:1	0.012	0.912
T43 BPT5204	Basta	49	36	13	3:1	0.055	0.814
T54 BPT5204	Basta	54	39	15	3:1	0.216	0.642
T63 BPT5204	Basta	29	21	8	3:1	0.096	0.756
T32 Chaitanya	BPH	60	45	15	3:1	0.000	-
T47 Chaitanya	BPH	35	26	9	3:1	0.010	0.920
T49 Chaitanya	BPH	43	32	11	3:1	0.007	0.933
T51 Chaitanya	BPH	30	22	8	3:1	0.041	0.839
T56 Chaitanya	BPH	31	23	8	3:1	0.010	0.920
T59 Chaitanya	BPH	39	29	10	3:1	0.059	0.808
T68 Chaitanya	BPH	31	23	8	3:1	0.010	0.920
T63 BPT5204	BPH	40	30	10	3:1	0.130	0.718
T32 Chaitanya	GLH	43	32	11	3:1	0.007	0.933
T47 Chaitanya	GLH	46	34	12	3:1	0.028	0.867
T49 Chaitanya	GLH	38	29	9	3:1	0.036	0.849
T51 Chaitanya	GLH	36	27	9	3:1	0.273	0.601
T56 Chaitanya	GLH	39	29	10	3:1	0.059	0.808
T59 Chaitanya	GLH	35	26	9	3:1	0.009	0.922
T68 Chaitanya	GLH	46	34	12	3:1	0.028	0.867
T63 BPT5204	GLH	39	29	10	3:1	0.059	0.808
T32 Chaitanya	WBPH	43	32	11	3:1	0.007	0.933
T47 Chaitanya	WBPH	50	37	13	3:1	0.026	0.871
T49 Chaitanya	WBPH	28	21	7	3:1	0.000	-
T51 Chaitanya	WBPH	49	36	13	3:1	0.055	0.814
T56 Chaitanya	WBPH	50	37	13	3:1	0.026	0.871
T59 Chaitanya	WBPH	38	28	10	3:1	0.036	0.849
T68 Chaitanya	WBPH	39	29	10	3:1	0.059	0.808
T63 BPT5204	WBPH	41	31	10	3:1	0.380	0.537

### Impact of ASAL on BPH, GLH and WBPH pests

**Figure 6:**
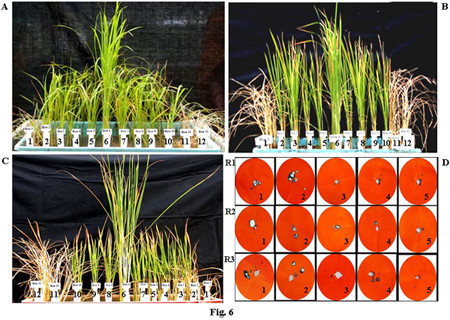
**Brown planthopper (BPH), green leafhopper (GLH) and whitebacked planthopper (WBPH) bioassays on T_2 _transgenic plants of Chaitanya and BPT5204 plants**. (A) 30-day old transgenic lines along with respective controls infested with BPH; (B) 30-day old transgenic lines along with respective controls infested with GLH; (C) 30-day old transgenic lines along with respective controls infested with WBPH; (D) Honeydew excretion by female BPH (R1: Row 1), GLH (R2: Row 2) and WBPH (R3: Row 3) insects after 24 hours of feeding on controls and transgenic rice plants; Rows 1 and 12: (var.TN-1) plants showing complete damage, Rows 2, 3, 4, 5, 7, and 8: Chaitanya transgenic lines showing resistance against BPH, GLH and WBPH, Row 6 A: Resistant check for BPH (var. PTB33), Row 6 B: Resistant check for GLH (var. Vikramarya), Row 6 C: Resistant check for WBPH (var. MO-1), Rows 9 and 10: BPT5204 transgenic lines showing resistance against BPH, GLH and WBPH, Row 11: Untransformed Chaitanya control plants. Photographs were taken after 14 days of infestation. D) 1 and 2 represent untransformed controls of Chaitanya and BPT5204, 3 and 4 represent T_49 _and T_51 _transgenic rice lines of Chaitanya, 5 represent T_63_transgenic rice line of BPT5204.

**Figure 7:**
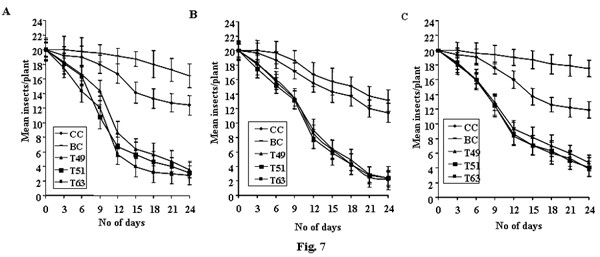
**Survival of BPH, GLH and WBPH insects on transgenic rice lines expressing ASAL**. Twenty 1^st^_- _instar nymphs of BPH (Figure A), GLH (Figure B) and WBPH (Figure C) were released on each plant on day 0. Homozygous transgenic lines T_49_, T_51 _and T_63 _are depicted by triangle, rectangle and square, respectively. Control plants are depicted by diamond and line. Bioassays were carried out on 20 plants sampled from each transgenic line and two controls. Differences between control and transgenic plants were significant at p < 0.005 from 6–24 days (ANOVA). Bars indicate mean ± SE.

Comprehensive *in planta *bioassay experiments were performed to test the insecticidal activity of the *asal *gene, on T_1 _and T_2 _(homozygous) transgenic lines, for three major sap-sucking pests of rice. Transgenic rice lines (30-day-old) expressing ASAL showed significant resistance towards BPH, GLH and WBPH insects with minimal plant damage (Fig. 6A, B and 6C). Transgenic plants exhibited varied levels (1–2 score on a 0–9 scale) of resistance to BPH, GLH and WBPH on a par with those of BPH-resistant var. PTB33, GLH resistant var. Vikramarya and WBPH resistant var. MO-1, respectively. On the other hand, susceptible var. TN-1 and untransformed control plants showed complete damage (9 score on a 0–9 scale) caused by these insects (Fig. 6A, B and 6C). Among eight T_2 _transgenic lines tested, two lines T_49 _and T_51 _of Chaitanya, and T_63 _of BPT5204, showed higher levels of resistance when compared to all other transgenic lines. The selected transgenic lines were further subjected to insect bioassays for mortality, developmental delay, fecundity and feeding behaviour of insects. ASAL-plants from selected lines, infested with BPH/GLH/WBPH nymphs, survived the infestation and could grow to maturity with normal seed set. The survival of BPH, GLH and WBPH nymphs fed on transgenic rice plants was reduced by ~74– 83%, ~79–84% and ~64–77%, respectively, as compared to that of susceptible control plants (Fig. 7A, B and 7C). During the entire 24-day bioassay period, the survival of BPH on transgenic rice plants was significantly reduced to 3.5 ± 1.1, 3.1 ± 1.6 and 2.8 ± 1.3 insects/plant on T_49_, T_51 _and T_63 _lines, respectively, compared to 12.4 ± 1.3, and 16.4 ± 1.6 insects/plant on untransformed control plants (Fig. 7A). Likewise, the survival of GLH on transgenic rice was significantly reduced to 2.4 ± 1.1, 2.4 ± 1.6 and 2.2 ± 1.1 insects/plant on T_49_, T_51 _and T_63 _lines, respectively, compared to 11.4 ± 1.3 and 13.4 ± 1.5 insects/plant on control plants (Fig. 7B). The survival of WBPH fed on transgenic plants was also reduced to 4.7 ± 1.1, 4.1 ± 1.3 and 3.9 ± 1.1 insects/plant on T_49_, T_51_, and T_63 _lines, respectively, in comparison with 11.9 ± 1.1 and 17.5 ± 1.2 insects observed on control plants (Fig. 7C).

### Entomotoxic effects of ASAL on sap-sucking pests

**Figure 8:**
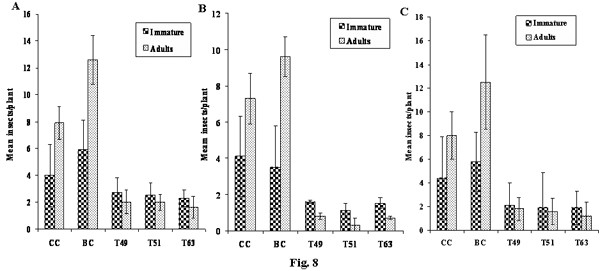
**Effect of ASAL on the development of BPH, GLH and WBPH insects**. Twenty 1^st^_- _instar nymphs of BPH (Figure A), GLH (Figure B) and WBPH (Figure C) were released on untransformed controls and transgenic plants on day 0, after 24 days the number of nymphs which reached adult stage and the number of nymphs which remained immature because of delay in development on control and transgenic lines were plotted on the graph. Bioassays were carried out on 20 plants sampled from each transgenic line and two controls. Differences between control and transgenic plants were significant at p < 0.005 (ANOVA). Bars indicate mean ± SE. CC: Chaitanya control plants, BC: BPT5204 control plants, T_49 _and T_51_: Chaitanya homozygous lines, T_63_: BPT5204 homozygous line.

First instar nymphs of BPH, GLH and WBPH were released onto transgenic and control rice plants and were monitored for the effect of ASAL on their growth and development. Insects fed on transgenic plants revealed ~10 to 12 days delay for reaching adulthood, as compared to the insects fed on untransformed control plants. Among BPH survivors, 10 to 27% could reach the adult stage on different transgenic lines, compared to 73 to 90% adults observed on control plants (Fig. 8A). From GLH survivors, only 10 to 15% could reach the adult stage on transgenic lines while 85 to 90% adults were found on control plants (Fig. 8B). In case of WBPH survivors, 18 to 30% could develop into adults on transgenics compared to 70 to 82% adults on control plants (Fig. 8C).

**Figure 9:**
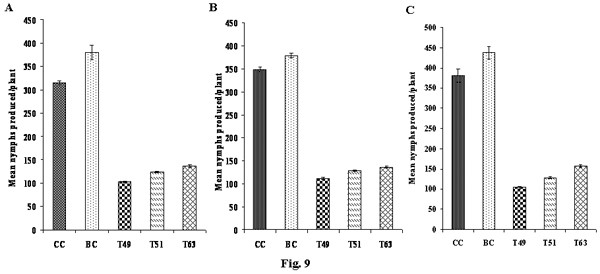
**Effect of ASAL on the fecundity of BPH, GLH and WBPH insects**. Total number of nymphs produced by a pair of adult BPH (Figure A), GLH (Figure B) and WBPH (Figure C) insects on controls and transgenic plants were counted and were plotted on the graph. Bioassays were carried out on 20 plants sampled from each transgenic line and controls. Differences between control and transgenic plants were significant at *p *< 0.005 (ANOVA). Bars indicate mean ± SE. CC: Chaitanya control plants, BC: BPT5204 control plants, T_49 _and T_51_: Chaitanya transgenic lines, T_63_: BPT5204 transgenic line.

Effect of ASAL on the fecundity of BPH, GLH and WBPH was assessed by estimating the total number of nymphs produced by the insects fed on transgenic rice plants. A mean number of 103 ± 3.9, 124 ± 2.7 and 137 ± 3.2 BPH nymphs/plant were recorded on T_49_, T_51 _and T_63 _plants compared to 315 ± 4.5 and 379.9 ± 15.6 nymphs/plant on untransformed controls (Fig. 9A). In case of GLH, a mean number of 112 ± 1.4, 121 ± 0.9 and 136 ± 2.2 nymphs/plant were observed on T_49_, T_51_, and T_63 _plants in comparison with 348 ± 5.01 and 378 ± 4.7 nymphs/plant on controls (Fig. 9B). Similarly, for WBPH a mean number of 104 ± 1.55, 128 ± 1.28 and 157 ± 2.21 nymphs/plant were noticed on T_49_, T_51 _and T_63 _plants as compared to 380 ± 5.2 and 438 ± 7.9 nymphs/plant produced on untransformed controls (Fig. 9C).

### Effect of ASAL on the feeding behaviour of BPH, GLH and WBPH insects

**Figure 10:**
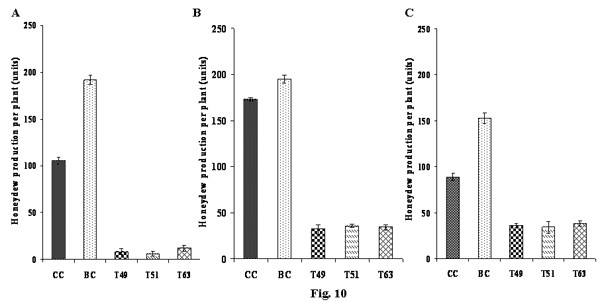
**Effect of transgenic plants expressing ASAL on feeding behaviour of BPH, GLH and WBPH insects**. A). Semi-quantitative estimation of honeydew excretion by BPH insects; B). Semi-quantitative estimation of honeydew excretion by GLH insects; C). Semi-quantitative estimation of honeydew excretion by WBPH insects; CC: Chaitanya control, BC: BPT5204 control, T_49 _and T_51_: Chaitanya transgenic lines, T_63_: BPT5204 transgenic line. Bars indicate mean ± SE.

The feeding ability of insects was assessed based on the amount of honeydew excreted by the insects. After a lapse of 24 h of feeding on transgenic rice/untransformed control plants, the number of honeydew units (blue spots) developed on the bromocresol green paper was counted to estimate the feeding capacity of the insects. A mean number of ~8 ± 1.36, ~21 ± 2.12 and ~25 ± 4.06 honeydew units/plant were excreted by BPH, GLH and WBPH, respectively, when fed on different transgenic rice plants compared to ~162 ± 6.7, ~173 ± 6.32 and ~189 ± 7.3 honeydew units/plant observed on control plants (Fig. 10A, B and 10C). Honeydew assay revealed significant reductions of ~92 to 94% and ~80 to 83% and ~60–68% in the feeding ability of BPH, GLH and WBPH, respectively, on transgenic plants as compared to the insects fed on control plants (Fig. 10A, B and 10C).

## Discussion

In our ongoing efforts to clone and introduce different plant lectin genes into rice genome against homopteran pests, we have been evaluating the potential usefulness of *A. sativum *agglutinin (*asal*) gene to provide resistance against major sap-sucking insects of rice, viz., BPH, GLH and WBPH which cause severe damage to rice plant affecting crop productivity. Earlier, we reported that transgenic rice engineered with the *gna *gene from *G. nivalis *could confer substantial resistance against major sap sucking pests [23,24]. An approximate 14% of the global cereal crop production is lost annually by the infestation of diverse insects. Among major cereals, about 83% of rice, 52% of wheat and 59% of maize crop production are lost owing to the damages caused by insect pests [42]. It has been estimated that >200 million tons of rice are being lost annually due to pests, despite intensive applications of chemical sprays to combat the menace [43]. Damages caused to various crops by sucking pests have become a serious concern, as they are difficult to control owing to their unique feeding strategy. Furthermore, after the introduction of *Bt *genes into various crops, the damages caused by sucking pests steadily increased as they failed to convey resistance against these pests. Several plant lectins have proved insecticidal against a wide array of pests belonging to lepidoptera, coleoptera, diptera and homoptera [20,21,23-25,37]. This study deals with the isolation of *A. sativum *lectin gene (*asal*) and its constitutive expression in two high-yielding indica rice cultivars. Further, strong entomotoxic effects of ASAL transgenics against major sap-sucking pests has been demonstrated employing standard screening techniques that reflect situations prevailing in the field.

The deduced amino acid sequence of ASAL protein disclosed maximum similarity (~98%) with that of previously reported garlic lectin protein [38,44]. Employing the protocols optimized in our laboratory, the co-integrated super-binary vector pSB111-*bar-asal *has been used to transform the elite indica rice cultivars. PCR and Southern analyses of BASTA tolerant plants confirmed the stable integration of *bar *and *asal *genes into the indica rice genome. Presence of ~1.6 kb hybridizable band with the *asal *probe in *Hind*III digested DNA, and ~1.9 kb hybridizable band with the *bar *probe in *Eco*RI digested DNA of transformants indicate the existence of two intact expression units of *bar *and *asal *in the rice genome (Fig. 3A and 3B). Similarly, *Eco*RI digested genomic DNA, when probed with *asal*, revealed a specific hybridizable band of >3.0 kb in different transformants (Fig. 3C), thereby suggesting the independent nature of transgene integration in primary transformants. These observations further suggest that the T-DNA is integrated into rice genome as a single copy without any rearrangement. It has been established that multiple copies of transgene(s) often result in co-suppression and gene silencing [45,46]. Single copy integration of transgene(s) is essential to achieve predictable patterns of inheritance and to eliminate the problem of gene silencing in transgenic plants [47]. Moreover, an inverse co-relation has been found between transgene copy number and their expression levels [48,45,49]. Northern blot analysis clearly showed the variable expression of *asal *gene in the primary transgenic plants as evidenced by varied intensity of the hybridizable band of >600 bp (Fig. 4). Western blot analyses of transgenic plants confirmed the stable expression of the *asal *gene at the protein level (Fig. 5). Marked variation observed in the amount of ASAL (0.74% to 1.45%), in different transformants by ELISA analysis, amply suggests that the transgene is integrated randomly at different transcriptionally active sites in the rice genome. The amount of ASAL expressed in different transgenic plants is distinctly higher as compared to the expression levels of GNA [32,23] and ASAL [38] proteins reported earlier.

To establish the definitive transgenic nature of primary transformants, the inheritance pattern of transgenes was analyzed in the T_1 _generation. BASTA test and Southern analysis indicated that *bar *and *asal *are transmitted in a Mendelian fashion. Segregation analyses of transgenes in T_1 _progenies revealed a monogenic ratio of 3 resistant: 1 susceptible plant(s) for both herbicide tolerance and insect resistance, affirming that these genes are stably integrated into the rice genome (Table. 1). The co-segregation of transgenes further confirms that both *bar *and *asal *are integrated and manifest as a single locus.

*In planta *insect bioassays amply indicate that expression of ASAL in transgenic rice lines imparts substantial resistance against BPH, GLH and WBPH insects, as evidenced by decreased insect feeding and declined insect survival, thereby minimizing the damage caused by hopperburn. The T_2 _progenies of eight independent homozygous transgenic lines (Fig. 6), subjected to insect bioassays, exhibited high-level resistance (1–2 score on a 0–9scale), testifying that ASAL affords ample protection against sap-sucking insects. The 1^st ^instar BPH/GLH/WBPH nymphs, when fed on selected T_2 _transgenic lines, viz., T_49_, T_51 _and T_63_, expressing 1.45%, 0.98% and 1.22% ASAL, respectively, disclosed ~50% mortality within 10–12 days of infestation (Fig. 7A, B and 7C). After 24 days of infestation, insects surviving on transgenic plants varied from 2 to 4/plant (Fig. 7) which exhibited delayed moulting and prolonged life cycle (~10 days) as compared to the insects present on susceptible control plants. The survival of BPH was reduced by ~74% on Chaitanya transgenic lines and ~83% on BPT5204 transgenic lines compared to the control plants (Fig. 7A). Similarly, GLH survival was decreased by ~79% on Chaitanya transgenic lines and ~84% on BPT5204 transgenic lines when compared to the controls (Fig. 7B). However, the survival of WBPH was declined by ~64% on Chaitanya transgenic lines and ~77% on BPT5204 transgenic lines in comparison with susceptible control plants (Fig. 7C). The accrued results amply indicate that the ASAL expressing transgenic rice exhibit a distinctly higher-level of resistance against both BPH and GLH pests as compared to that of GNA [23] and ASAL [38] expressing rice lines. It was reported that overexpression of ASAL and GNA in rice reduced the survival of BPH by 36% [38] and 32% to 59%, respectively [32,50,51,23,12]. Similarly, the GLH survival was decreased by 49% to 53% on GNA transgenics [5,23,12] and by 32% on ASAL expressing plants [38]. Whereas, WBPH nymphs fed on ASAL transgenic rice showed ~64–77% mortality as compared to 90% mortality observed on GNA transgenics [24]. Furthermore, fecundity assays conducted on ASAL rice lines revealed significant decline in the nymphal production of BPH, GLH and WBPH insects by ~68%, ~73% and ~67% on Chaitanya transgenic lines and ~64%, ~65% and ~64% on BPT5204 transgenic plants, respectively (Fig. 9A, B and 9C), indicating marked decreases in the fecundity of BPH, GLH and WBPH insects. These results suggest the high antifeedant and entomotoxic effects of ASAL on these insects. A clear correlation has been observed between the amount of garlic lectin in transgenic plants and its entomotoxic effects on three sap-sucking insects. Earlier, it was reported that BPH and GLH nymphal production was reduced by 59% and 70.5% when fed on ASAL expressing transgenic rice [38]. However, transgenic rice expressing GNA showed a striking decrease in the fecundity (~90%) of WBPH [24] as compared to the ASAL transgenics (~66%) employed in this study. Also, marked decreases of ~92 to 94% and ~80 to 83% were observed in the honeydew production of BPH and GLH insects, respectively, when fed on ASAL transgenic plants (Fig. 10A, B and 10C), compared to the honeydew produced by the insects fed on GNA transgenics [23]. Conversely, GNA transgenics showed ~90% reduction in the honeydew production of WBPH [24] compared to ~60–68% reduction observed on ASAL transgenics. An overview of insect bioassays amply establish that ASAL is more toxic to both BPH and GLH insects compared to GNA; whereas, GNA showed higher toxicity to the WBPH than ASAL under similar bioassay conditions. The variable entomotoxic effects of GNA and ASAL on three sap-sucking pests may be attributed to their differential binding affinities to receptor proteins on gut epithelial cells of the insects. As it is imperative to identify eco-friendly and potent insecticidal proteins, the *asal *may be preferred for genetic engineering of diverse crops against sucking pests. The overall results of *in planta *insect bioassays establish that *asal *transgenic rice plants could express functionally active ASAL protein, which is comparable to the native form of garlic lectin(s) used in artificial diet bioassays against sucking insects [29,25].

Although the precise mechanism of lectin toxicity to insects is unclear, yet it probably involves binding of lectins to the receptors present on the gut epithelial cells of various insects [52]. Immunohistochemical studies of a wide range of mannose or mannose/glucose specific lectins such as GNA, Con A and PSA suggest the binding to the midgut epithelial cells of insects thereby contributing to the insecticidal effect [53]. Furthermore, the bound lectins might inhibit the absorption of nutrients or disrupt the midgut cells through endocytosis of lectin and other toxic metabolites [19]. The toxicity of mannose binding lectins towards sucking insects is not clear, but it has been shown to bind to the mannose moiety of brush border membrane vesicle (BBMV) receptors of gut epithelial cells, thereby causing disruption of cell function and mortality [54,55]. Banerjee et al. [56], using ligand blot analysis of the mustard aphid (BBMV), demonstrated that ASAL protein binds to symbionin (Sym L) receptor involved in the transmission of viruses by sucking pests.

## Conclusion

*In planta *insect bioassays, reported herein, were carried out on *asal *transgenic rice lines adopting standard screening techniques followed in the conventional rice breeding for selection of insect resistant plants. The ASAL expressing plants, bestowed with high antifeedant and antimetabolic effects, afforded high-level resistance against three major sap-sucking insects. T_4 _transgenic lines of Chaitanya and BPT5204 varieties are being evaluated in limited open-field trials in the hopper-prone areas. To our knowledge, none of the rice cultivars thus far developed by conventional methods could show worthwhile resistance against three major sap-sucking pests. The overall results amply indicate that *asal *transgenic rice lines exhibit surpassing resistance against BPH, GLH and WBPH insects. The prototypic transgenic rice harbouring exotic *asal *appear promising for direct commercial cultivation besides serving as a novel genetic resource in recombination breeding.

## Methods

### RNA extraction from garlic plants

Four-week-old garlic plants (var. Godavari) grown in the net house, at CPMB, O.U., were used for total RNA isolation. Plant material was ground in the liquid nitrogen and homogenized in an equivalent volume (w/v) of denaturing buffer (4 M guanidium thiocyanate, 25 mM sodium citrate (pH 7.0), 0.5% SDS, 0.1 M β-mercaptoethanol, 2 M sodium citrate pH 4.0, water saturated phenol and chloroform) [57]. The RNA was extracted with water saturated phenol, and was precipitated with 2 M sodium acetate and 2.5 volumes of ethanol. Later, RNA was dissolved in DEPC-treated water and reprecipitated with 4 M LiCl, and the same was redissolved in DEPC-treated water. The quality of RNA was checked on denaturing 1.4% agarose gel and quantified using spectrophotometer. From the total RNA, mRNA was obtained using the mRNA purification kit [58] as per manufacturers' instructions.

### Synthesis of cDNA and isolation of garlic lectin encoding gene (asal)

First strand cDNA was synthesized with Super Script II RNase H^- ^reverse transcriptase (200 U/μl, GIBCO-BRL) according to the manufacturer's protocol. The coding sequence of *asal *was obtained by PCR using primers 5'-GGA TTC ATG GGT CCT ACT ACT TCA TCT CCT-3', and 5'-GAA TTC TCA AGC AGC ACC GGT GCC AAC CTT-3', employing first strand cDNA as the template. The 25 μl PCR reaction mixture containing template DNA (100 ng), primers (10 μM), buffers, dNTPS (0.5 mM) and *pfu *DNA polymerase, was subjected to initial denaturation (94°C) for 5 min; followed by repeated denaturation (94°C) for 45 s, annealing (63°C) for 45 s, and elongation (72°C) for 1 min for a total of 35 cycles on PTC-200 Peltier Thermal Cycler. Final elongation was carried out at 72°C for 10 min. Amplified products were analyzed by gel electrophoresis on 1.0% agarose gel. PCR product was digested with *Bam*HI and *Eco*RI then ligated into pGEM-4Z [59] at *Bam*HI and *Eco*RI sites using the rapid ligation kit [60] and transformed into *E. coli *(Top10) cells. The recombinant clones were subjected to DNA sequencing using automated DNA sequencer.

### Construction of Ti-super binary vector containing asal and bar expression cassettes

The *asal *gene was excised with *Bam*HI and *Eco*RI enzymes from pZEM4Z vector, and cloned between CaMV35S promoter and *nos *terminator of intermediate vector pSB11 *bar *constructed earlier in our laboratory. The binary vector contains *bar *(CaMV35S-*bar*-*nos*) gene as a plant selection marker [61]. The recombinant vector, pSB11*bar*-CaMV35S-*asal-nos*, was maintained in HB101 cells and mobilized into *A. tumefaciens *strain LBA4404 by triparental mating [62] using the helper vector pRK2013 and the resulting co-integrate vector was designated as pSB111-*bar*-*asal*.

### Agrobacterium-mediated transformation and regeneration of transgenic plants

*Agrobacterium*-mediated genetic transformation experiments were carried out using LBA4404 strain harbouring pSB111-*bar*-*asal *super-binary vector. Two leading indica rice cultivars, Chaitanya and BPT5204, obtained from the Directorate of Rice Research (DRR), Hyderabad, were employed for genetic transformation. Mature seeds were manually dehusked and surface-sterilized with 0.1% (w/v) HgCl_2 _for 7 min followed by three washings with autoclaved distilled water, and kept at 29°C for germination. After 24 h of incubation, sprouted embryos were cut aseptically and placed on MS [63] medium (3MN62; MS basal + 30 g/l maltose + 2 mg/l 2, 4-D + 1 g/l casaminoacids + 50 mg/l tryptophan +100 mg/l Inositol + 0.3% gelrite) for callus induction. After 3 weeks of incubation, the scutellum-derived calli were used for transformation experiments. *Agrobacterium *cultures were initiated by inoculating a single colony of the bacterium into 6 ml YEP medium containing 50 mg/l spectinomycin and 10 mg/l tetracycline at 225 rpm and 29°C for 24 h. The bacterial culture was pelleted at 3500 rpm and resuspended in 10 ml of PIMII medium [39] supplemented with 100 μM acetosyringone, and incubated for 16 h at 29°C. Before co-cultivation, the embryogenic calli were cut into small pieces, and were treated with MS basal medium supplemented with 100 mM acetosyringone for 30 min. Later, calli were transferred into the *Agrobacterium *culture and left on the shaker at 225 rpm for 30 min. These calli were placed on the co-cultivation medium and 20 μl of *Agrobacterium *culture was added on each callus for infection [12]. Infected calli were incubated for 72 h at 29°C in dark and washed thrice in MS basal supplemented with 250 mg/l cefotaxime and 100 mg/l carbenicillin, and kept in 3MN62 medium containing the above antibiotics for 2 weeks. Proliferated calli were subjected to two rounds of selection containing 8 mg/l and 10 mg/l phosphinothricin for four weeks [23,12]. After 4 weeks of incubation on selection medium, the surviving calli were selected and cultured on the proliferation medium [12] for 2 weeks. Later, actively growing calli were transferred to the regeneration medium containing BAP (3–4 mg/l) and NAA (0.1–0.5 mg/l). Subsequently, the regenerated shoots were transferred onto the 1/2 MS rooting medium, and rooted plants were transferred into pots and grown to maturity in the glasshouse. Transgenic plants (30–40 day-old) along with untransformed controls were tested for their tolerance to the herbicide BASTA [61].

### Southern blot analysis

Genomic DNA was isolated from the BASTA tolerant and untransformed control plants using the method of [64]. PCR analysis was carried out using the primers corresponding to the genes *asal *(5'-ATG GGT CCT ACT ACT TCA TCT CCT-3'; 5'-TCA AGC AGC ACC GGT GCC AAC CTT-3') and *bar *(5'-CTA CCA TGA GCC CAG AAG G-3'; 5'-TCA GAT CTC GGT GAC GGG-3'). The DNA from the untransformed control plants was used as negative control and the intermediate vector was used as positive control. For Southern blot analysis [57], approximately 10–12 μg of genomic DNA was digested with *Eco*RI and *Hind*III separately, electrophoresed on a 0.8% agarose gel and subsequently transferred to an N^+ ^Nylon membrane [58] and fixed by exposing to UV (1200 μJ for 60 s) in an UV cross linker. DNA blot was pre-hybridized with sodium phosphate buffer (pH 7.2) containing 7% SDS and blocking reagent (Salmon sperm DNA) at 65°C for 6 h. Hybridization was carried out with the same buffer at 65°C for 18–20 h. The 546 bp *asal *and 560 bp *bar *coding regions were used as probes after labelling with α-^32^P dCTP employing ready to go random primer DNA labelling kit [58]. The membrane was washed at room temperature (37°C) twice in buffer 1 (2 × SSC+0.1%SDS) for 20 min each, followed by once in buffer 2 (1 × SSC+0.1% SDS) for 15 min at 65°C and once in buffer 3 (0.1 × SSC+0.1%SDS) for 10 min at 65°C. Later, membranes were exposed to X-ray film for 24–48 h at -70°C.

### Northern blot analysis

Northern blot analysis was carried out according to [57]. About 20 μg of total RNA was separated on 1.4% denaturing agarose gel and was blotted onto nylon membrane and fixed by exposing to UV (1200 μJ for 60 s) in an UV cross linker. Pre-hybridization, hybridization and washing steps were carried out as described above for Southern blot analysis.

### Western blot analysis

Samples of transgenic and untransformed control leaf tissue were homogenized in 50 mM Tris-HCl buffer pH 9.0. The extract was centrifuged at 5000 g for 20 min at 4°C, and the supernatant was collected. Protein samples (5 μg) were subjected to 15% SDS-PAGE according to [65]. Following electrophoresis, the separated proteins were transferred onto nitrocellulose N^- ^membrane [58] by electroblotting [66]. After protein transfer, the membrane was blocked by incubating in PBS solution containing 10% non fat dried milk and 0.1% Tween 20 for 2 h at room temperature. The membrane was probed with polyclonal rabbit anti-*asal *serum (1:10000 dilution) and goat anti-rabbit IgG horse-radish peroxidase conjugate [67] as secondary antibody (1:10000 dilution). The membrane was washed and revealed with saturated benzidine solution containing 20% ammonium chloride and 0.1% H_2_O_2_.

### ELISA analysis

Wells of the microtitre plate were coated with 1 μg of crude protein extract of transgenic plants and kept for overnight at 37°C and at 4°C for 1 h. The wells were washed thrice with 20 mM PBS containing 0.05% Tween 20 and were blocked with 10% non-fat dried milk for 2 h at 37°C, subsequently washed six times with PBS-T. The primary antibody (1:10000) was added to the wells and incubated for 2 h at 4°C. After incubation, the wells were washed thrice with PBS and incubated with secondary antibody (1:10000) for 1 h at room temperature. The plates were washed thrice with PBS and 0.001% TMB substrate in 0.05 M phosphate citrate buffer was added along with 0.1% H_2_O_2 _and kept in dark for 10 min. The reaction was stopped by 1 N H_2_SO_4 _and the absorbance was recorded on ELISA reader at 450 nm.

### Insect bioassays

Insect bioassays of brown planthopper (BPH), green leafhopper (GLH) and white backed planthopper (WBPH) were carried out on both T_1 _and T_2 _*asal *transgenic plants along with their respective controls as well as susceptible control Taichung Native 1 (TN-1) and resistant checks PTB33, Vikramarya and MO-1 at the Directorate of Rice Research, (DRR), Rajendranagar, Hyderabad. All bioassays were carried out at 25°C under an approximate 16 h/8 h light/dark photoperiod regime. The BPH, GLH and WBPH insects were maintained on 25–30 day old TN-1 plants under controlled conditions in the glass house. Premated gravid females of BPH, GLH and WBPH were allowed to ovi-posit separately on TN-1 plants for two days and the freshly hatched nymphs or the nymphs after attaining the desired age were utilized for various experiments. The degree/level of resistance exhibited by transgenic rice plants was scored based on a scale of 0–9, as used in the International rice testing programme [23]. Homozygous transgenic lines T_49_, T_51 _of Chaitanya, and T_63 _of BPT5204, expressing high levels of ASAL were employed for developmental and fecundity assays of BPH, GLH and WBPH insects. In these experiments each plant was confined in an insect proof clean plastic cylinder (50 cm in length and 15 cm in diameter) around the stem of the plant and top of the plastic cylinder was sealed with fine nylon mesh. For developmental assay each of the 20 BPH, GLH and WBPH first instar nymphs were introduced separately on each plant confined in an insect proof cage and mortality of insects was monitored at 3 day intervals for 24 days [23]. Twenty replicates were kept for each treatment. For the fecundity assay, the male and female insects were confined together in a 1 male: 1 female ratio to avoid differences in the nymphal production based on sex ratio. The number of nymphs produced from the eggs was counted until no new nymphs were found emerging. Data were analyzed using the sigma plot software, version 5.0, for windows (SPSS, Richmond, Calif., USA). Differences between the mean values were subjected to unpaired *t*-test or ANOVA.

### Honeydew (liquid excreta) assay

The extent of insect feeding was also estimated by semi-quantitative assay of the honeydew produced [68]. Whatman no.1 filter paper dipped in a solution of bromocresol green (2 mg/ml in ethanol) was used for honeydew estimation. The filter paper was placed at the base of each plant and covered with a plastic cup. On each plant five female adult insects of BPH, GLH and WBPH pre-starved for 2 h, were released separately and allowed to feed for 24 h. GLH feeding was confined to the leaf blades by placing the filter paper at the base of leaves. Insect excreta (honeydew) react with the bromocresol green on the filter paper resulting in blue colour spots. The area of blue spots that developed on the filter paper was measured using millimeter graph paper and expressed in units (1 unit = 1 mm^2^) as per [23].

## Authors' contributions

YB and SVK carried out the isolation, cloning of the gene, genetic transformation, molecular characterization of transgenics and insect bioassays on transgenic plants; ICP designed and assisted in carrying out insect bioassays; VDR and KVR designed the strategy, data analysis and interpretation, and drafted the manuscript.
